# Characterizing directed functional pathways in the visual system by multivariate nonlinear coherence of fMRI data

**DOI:** 10.1038/s41598-018-34672-5

**Published:** 2018-11-05

**Authors:** Gadi Goelman, Rotem Dan, Tarek Keadan

**Affiliations:** 1 Department of Neurology, Hadassah Hebrew University Medical Center, Jerusalem, Israel; 2 Edmond and Lily Safra Center for Brain Sciences (ELSC), the Hebrew University of Jerusalem, Jerusalem, Israel

## Abstract

A multivariate measure of directed functional connectivity is used with resting-state fMRI data of 40 healthy subjects to identify directed pathways of signal progression in the human visual system. The method utilizes 4-nodes networks of mutual interacted BOLD signals to obtains their temporal hierarchy and functional connectivity. Patterns of signal progression were defined at frequency windows by appealing to a hierarchy based upon phase differences, and their significance was assessed by permutation testing. Assuming consistent phase relationship between neuronal and fMRI signals and unidirectional coupling, we were able to characterize directed pathways in the visual system. The ventral and dorsal systems were found to have different functional organizations. The dorsal system, particularly of the left hemisphere, had numerous feedforward pathways connecting the striate and extrastriate cortices with non-visual regions. The ventral system had fewer pathways primarily of two types: (1) feedback pathways initiated in the fusiform gyrus that were either confined to the striate and the extrastriate cortices or connected to the temporal cortex, (2) feedforward pathways initiated in V2, excluded the striate cortex, and connected to non-visual regions. The multivariate measure demonstrated higher specificity than bivariate (pairwise) measure. The analysis can be applied to other neuroimaging and electrophysiological data.

## Introduction

Understanding the organization of large scale neural networks is essential for the understanding of brain function^1^. For this purpose, neuroimaging data of MRI, EEG and MEG can be used to construct structural (anatomical links), functional or effective connectivity macroscopic scale networks^2^. Directed functional connectivity refers to methods that appeal to temporal precedence to infer directed connectivity^3^. These include Granger causality^4^, phase-transfer entropy^5^ and the multivariate method we have recently introduced that is based upon nonlinear coherences among multiple time-series and systematic phase delays between the regions^6^. Here, we used an elaboration of this method to identify pathways of signal progression within the visual system and compare characteristics of the dorsal and ventral streams. For these purposes we extended the method and introduced a statistical framework that enables to obtain model-free identification of signal progression patterns. By assuming a fixed relationship between neuronal and hemodynamic phases and a constant hemodynamic response (delays) across the measured systems, our method infers patterns of neural information flow. In contrast to other functional connectivity methods that are based on temporal similarity and connectivity strength (i.e. amplitudes), our method is based on temporal hierarchy between multiple time-series (i.e., the temporal order of signal progression) and on nonlinear coherence between them (i.e. phases). For the application of the method to functional MRI (fMRI) data, there is a need to assume that the hemodynamic lag faithfully reproduces temporal precedence at the neuronal level. Based on our^7^ and others results, we will make this simplifying assumption to demonstrate the potential usefulness of our method for fMRI. Previous fMRI studies^8–11^ showed that time-lag propagates within conventionally known resting-state networks and therefore can be used to infer MRI signal progression and its directionality^10,11^.

Cortical visual processing is commonly thought to proceed along two distinct pathways: a fast, highly recurrent, dorsal pathway projecting into the parietal cortex and a slower ventral pathway projecting into the inferior temporal cortex. These pathways were identified in monkeys as anatomically and functionally distinct systems of multisynaptic connections emerging from the striate cortex^12,13^. The initial conception of the ventral stream was that of a serial hierarchical pathway starting at the striate cortex (area V1), passing through a sequence of processing stages in the extrastriate cortex until complex object representations are formed in the anterior part of the inferior temporal cortex (area IT)^14,15^. A more recent view based on electrophysiology measurements in macaques, claims that the ventral visual pathway is a recurrent and highly interactive occipitotemporal network linking early visual areas and the anterior IT cortex along multiple routes through which visual information is processed^13^. Likewise, the original notion of the serial processing stages in the dorsal stream was challenged by recent findings suggesting several distinct pathways that emerge from this stream^12^. Furthermore, previous studies have shown that the dorsal system is even more complex. For example, earlier activations in MT than in V1 have been demonstrated^16^. Such findings suggest that the extrastriate cortex in the dorsal but not in the ventral stream is activated early enough to be able to influence subsequent processing through feedback connections to V1^17^.

Here, we applied our directed functional connectivity method to resting-state fMRI data of 40 healthy young subjects. We aimed to test whether the pathways obtained in the visual system using resting-state data are in line with previous studies that mainly were obtained by stimulus driven data. Such findings will suggest common patterns of resting-state and stimulus driven data and on the other hand will validate the application of our method to fMRI data. Furthermore, based on the literature described above demonstrating different organizations of the ventral and dorsal streams, we aimed to capture these differences correctly, such that it would be possible to detect distinct patterns of coupling in normal subjects or, perhaps, aberrant patterns or disconnections in psychiatric disorders^18^.

## Mathematical Methodology

Recently we have shown that for a network composed of 4 coupled time-series, the network’s functional connectivity strength (i.e. amplitude) and the relations between the 4 time-series phases (i.e. coherence) can be analytically described as a function of time and frequency^6^. Using wavelet analysis we have shown that for each time-frequency point there are three distinct forms of interactions between the 4 coupled time-series, each corresponding to a different coherence. The full interaction can be obtained (but used here only for comparison with bivariate strength, see below) by multiplying the 3 distinct expressions. The validity of the full interaction expression can be tested, for example, in the case where time-series 1 and 2 are equal and so are time-series 3 and 4. In this case, the full interaction converges to pairwise coherence as expected. The three distinct coherences (termed in hereafter “functional connectivity”, FC) equal:

1 $$\begin{array}{rcl}F{C}_{j1,j2,j3,j4}^{1}(\omega ,t) & = & ({W}_{\omega ,t}^{j1})\cdot {({W}_{\omega ,t}^{j2})}^{\ast }\cdot ({W}_{\omega ,t}^{j3})\cdot {({W}_{\omega ,t}^{j4})}^{\ast }\\  & = & A\cdot \{\exp [i({\vartheta }_{\omega ,t}^{j1}-{\vartheta }_{\omega ,t}^{j2}+{\vartheta }_{\omega ,t}^{j3}-{\vartheta }_{\omega ,t}^{j4})]\}=A{e}^{i{\phi }^{a}}\\ F{C}_{j1,j2,j3,j4}^{2}(\omega ,t) & = & ({W}_{\omega ,t}^{j1})\cdot ({W}_{\omega ,t}^{j2})\cdot {({W}_{\omega ,t}^{j3})}^{\ast }\cdot {({W}_{\omega ,t}^{j4})}^{\ast }\\  & = & A\cdot \{\exp [i({\vartheta }_{\omega ,t}^{j1}+{\vartheta }_{\omega ,t}^{j2}-{\vartheta }_{\omega ,t}^{j3}-{\vartheta }_{\omega ,t}^{j4})]\}=A{e}^{i{\phi }^{b}}\\ F{C}_{j1,j2,j3,j4}^{3}(\omega ,t) & = & ({W}_{\omega ,t}^{j1})\cdot {({W}_{\omega ,t}^{j2})}^{\ast }\cdot {({W}_{\omega ,t}^{j3})}^{\ast }\cdot ({W}_{\omega ,t}^{j4})\\  & = & A\cdot \{\exp [i\,({\vartheta }_{\omega ,t}^{j1}-{\vartheta }_{\omega ,t}^{j2}-{\vartheta }_{\omega ,t}^{j3}+{\vartheta }_{\omega ,t}^{j4})]\}=A{e}^{i{\phi }^{c}}\end{array}$$

where, ${W}_{\omega ,t}^{j1}$ is the wavelet coefficient in time and frequency of time-series ‘*j1*’, ${\vartheta }_{\omega ,t}^{j1}$ is the phase of time-series ‘*j1*’, *‘A’* is the coherence amplitude, *denotes the complex conjugate and *‘i’* is the imaginary unit. The nonlinear coherence phases*, φ*^*a*^*, φ*^*b*^ and *φ*^*c*^, can be computed according to Equation 1 and were used to obtain the phases of the time-series for each frequency and time point^6^:

2 $$\begin{array}{c}\begin{array}{ccc}{\vartheta }_{\omega ,t}^{j2}={\vartheta }_{\omega ,t}^{j1}\mp \frac{{\phi }^{a}+{\phi }^{c}}{2}; & {\vartheta }_{\omega ,t}^{j3}={\vartheta }_{\omega ,t}^{j1}\mp \frac{{\phi }^{b}+{\phi }^{c}}{2}; & {\vartheta }_{\omega ,t}^{j4}={\vartheta }_{\omega ,t}^{j1}\mp \frac{{\phi }^{a}+{\phi }^{b}}{2}\end{array}\\ \begin{array}{ccc}{\vartheta }_{\omega ,t}^{j2}={\vartheta }_{\omega ,t}^{j3}\mp \frac{{\phi }^{a}-{\phi }^{b}}{2}; & {\vartheta }_{\omega ,t}^{j4}={\vartheta }_{\omega ,t}^{j3}\mp \frac{{\phi }^{a}-{\phi }^{c}}{2}; & {\vartheta }_{\omega ,t}^{j4}={\vartheta }_{\omega ,t}^{j2}\mp \frac{{\phi }^{b}-{\phi }^{c}}{2}\end{array}\end{array}$$

where $\mp$ is due to the freedom in the phase definition which requires specification of the sign^6^. Following the logic that phases correspond to time-lags at each frequency and time points, Equation 2 can be used to define the hierarchical temporal order among the 4 time-series. Note that for the definition of directed functional connectivity given below, only the phases (Equation 2) were used and not the amplitude of functional connectivity. This is in line with the definition of related nonlinear coupling measures that are based on phase relationships, such as phase dispersion or phase locking analyses. Note that the phase differences of Equation 2 are different from the corresponding pairwise phase-differences since they include the effect of the other time-series as well.

To fully characterize a network of 4 time-series, the network’s temporal order of the time-series needs to be identified, namely the order of signal propagation among the time-series which is given by the temporal pathway that describes how the signal is propagating throughout the network. Acknowledging that the phases of the time-series (at each frequency) are related to time-delays, the phases can be used to outline a unique ordinal or hierarchical ranking of signal progression. A network of 4 time-series has 24 ( = 4!) possible pathways (listed in Table 1). Using the phases to define ordinal ranking requires to assume that all phases are below 2π.For example, consider the signal progression pathway: R1 → R2 → R3 → R4, where R1 to R4 represent the 4 time-series. If we use the phase of R1 as a reference, Equation 2 can be used to calculate the three phase-differences between the time-series: $(({\vartheta }_{2}-{\vartheta }_{1});({\vartheta }_{3}-{\vartheta }_{1});({\vartheta }_{4}-{\vartheta }_{1}))$. If $({\vartheta }_{4}-{\vartheta }_{1}) > 2\pi$, the pathway can mistakenly be identified as R4 → R1 → R2 → R3. This demonstrates a bias in pathway identification. It also shows that pathway definition depends on the reference phase. To overcome these biases, we introduce the concept of “Circular Pattern” (CP). A circular pattern defines how signals propagate within the network. For example we note that pathways: R1 → R2 → R3 → R4, R2 → R3 → R4 → R1, R3 → R4 → R1 → R2 and R4 → R1 → R2 → R3 have all the same pattern of signal progressing but are differ in their starting point within this pattern. Circular-patterns are invariant to the choice of reference phase, and are less sensitive to phase values greater then 2*π*. We note that a CP does not give the temporal order of the time series but only its pattern of signal progression. To obtain a pathway from a CP, an assumption for the starting point has to be made. Table 1 show that for a network of 4 time-series there are six possible CPs, where each CP includes 4 different pathways. We note that the six CPs can be divided into three pairs (CP1 and CP4; CP2 and CP3; CP5 and CP6) where each CP in the pair is of an opposite flow pattern. These pairs are the consequence of the intrinsic directional freedom in the definition of phases that mathematically is expressed by the $\mp$ sign in Equation 2. Note that the above CP analysis can be performed for each time-frequency point, therefore enabling one to characterize the dynamics of signal progression. In other words, one can in principle evaluate circular patterns instantaneously at any desired frequency. This enables to track dynamic changes in the hierarchical patterns of coupling. We will address this elsewhere.

**Table 1:** The six Circular Patterns (CPs) presented as squares with lines corresponding to flow organization among the four time-series (R1 to R4).

CP 1	CP 2	CP 3	CP 4	CP 5	CP 6
$$\begin{array}{ccc}{\boldsymbol{R}}1 & - & {\boldsymbol{R}}2\\ | & & |\\ {\boldsymbol{R}}4 & - & {\boldsymbol{R}}3\end{array}$$	$$\begin{array}{ccc}{\boldsymbol{R}}1 & - & {\boldsymbol{R}}2\\ | & & |\\ {\boldsymbol{R}}3 & - & {\boldsymbol{R}}4\end{array}$$	$$\begin{array}{ccc}{\boldsymbol{R}}1 & - & {\boldsymbol{R}}3\\ | & & |\\ {\boldsymbol{R}}2 & - & {\boldsymbol{R}}4\end{array}$$	$$\begin{array}{ccc}{\boldsymbol{R}}1 & - & {\boldsymbol{R}}4\\ | & & |\\ {\boldsymbol{R}}2 & - & {\boldsymbol{R}}3\end{array}$$	$$\begin{array}{ccc}{\boldsymbol{R}}1 & - & {\boldsymbol{R}}3\\ | & & |\\ {\boldsymbol{R}}4 & - & {\boldsymbol{R}}2\end{array}$$	$$\begin{array}{ccc}{\boldsymbol{R}}1 & - & {\boldsymbol{R}}4\\ | & & |\\ {\boldsymbol{R}}3 & - & {\boldsymbol{R}}2\end{array}$$
R1-R2-R3-R4	R1-R2-R4-R3	R1-R3-R4-R2	R1-R4-R3-R2	R1-R3-R2-R4	R1-R4-R2-R3
R2-R3-R4-R1	R2-R4-R3-R1	R3-R4-R2-R1	R2-R1-R4-R3	R2-R4-R1-R3	R2-R3-R1-R4
R3-R4-R1-R2	R3-R1-R2-R4	R4-R2-R1-R3	R3-R2-R1-R4	R3-R2-R4-R1	R3-R1-R4-R2
R4-R1-R2-R3	R4-R3-R1-R2	R2-R1-R3-R4	R4-R3-R2-R1	R4-R1-R3-R2	R4-R2-R3-R1

In this illustration, flow is possible only along the lines connecting pairs of time-series. The four pathways corresponding to the CPs are listed below each CP. These pathways have the same flow pattern but they are different in their starting point. Note that CP1 and CP4; CP2 and CP3; and CP5 and CP6 correspond to pairs of opposite flow.

To test for the significance of the CPs, we adopted the common approach used in coherence studies. A group expectation value was calculated for each CP. This expectation value was named “Circular-Pattern Index” (CPI) and is defined on the phases as:

3 $$CP{I}^{k}=\frac{1}{N}{\sum }_{i=1}^{N}\{\begin{array}{c}1\\ 0\end{array}\}\begin{array}{c}phases\,in\,line\,with\,circular\,pattern\\ phases\,not\,in\,line\,with\,circular\,pattern\end{array}$$

where ‘k’ is a specific CP (k = 1, 2, 3, 4, 5 or 6). In Equation 3, it is tested for each subject separately whether the 4 time-series network’s phases are in line with the CP, i.e. if they are consistent with one of the four pathways which characterize the CP (Table 1). The calculations for the 6 CPs (Equation 3) are all done according to the same selection of phase direction in Equation 2. This selection reduces the degeneracy of the CP pairs. Equation 3 is defined similarly to the definition of the Phase Locking Value (PLV)^19–21^ or the Phase Lag Index (PLI)^22,23^. Namely, for each subject and each CP, a value of 1 is assigned when the network phases are in agreement with this CP, and zero is assigned when there isn’t an agreement. Consequently, if the probability to obtain a specific CP ‘k’ among the N subjects is low, the CPI^*k*^ will be close to zero. On the other hand, for a high probability of obtaining this CP, CPI^*k*^ will be high and approach unity. Permutation-based non-parametric tests with uncoupled time-series were used to obtain the CPI null distributions for each CP at each frequency and assign a *p*-value for certain CPI^*k*^ which is defined in terms of pattern-specific indicator functions.

**Figure 1:**
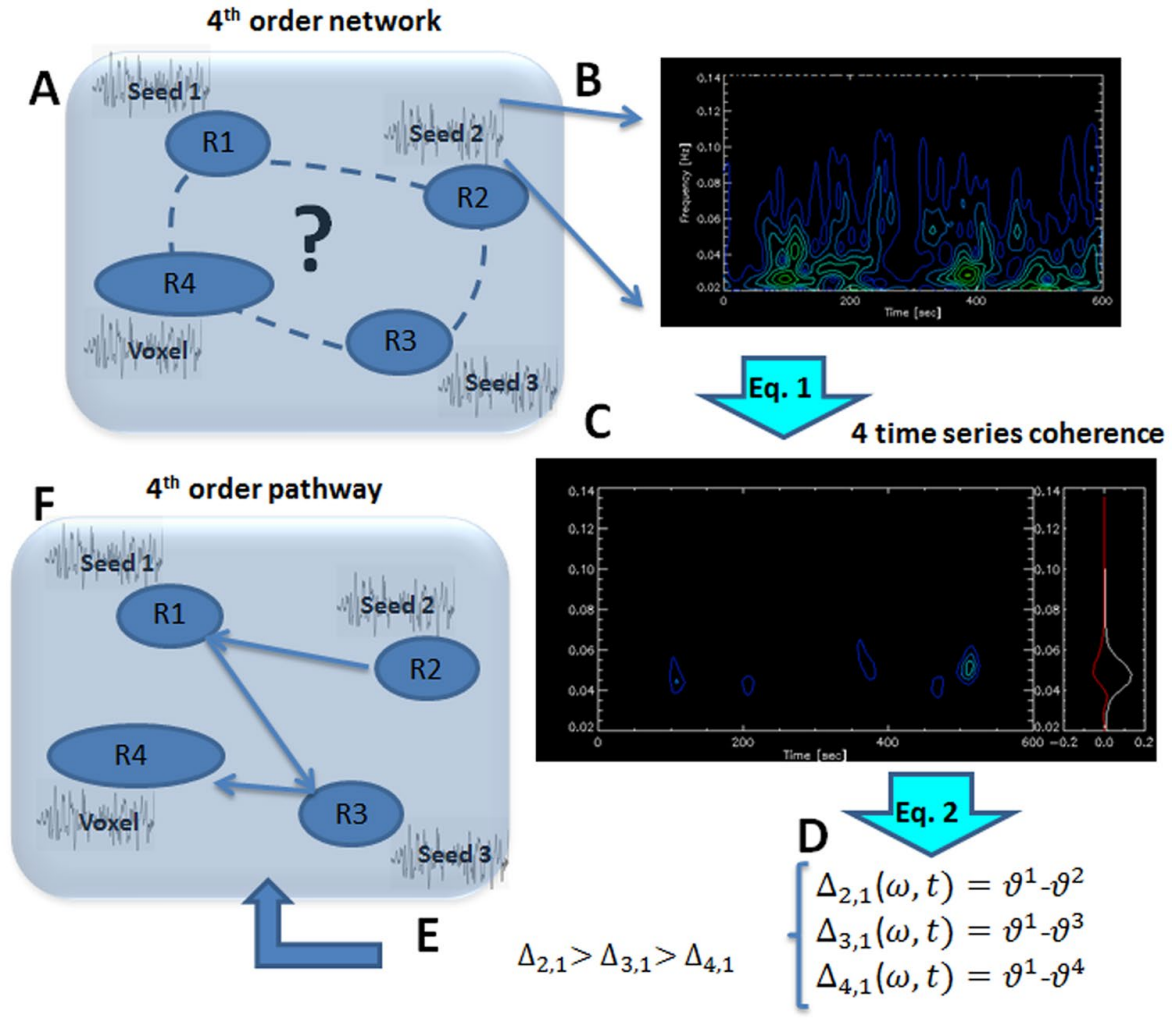
An illustration of the process applied to define directed multivariate functional pathways in a 4 time-series network. (**A**) A network of 4 time-series (R1, R2, R3 and R4), where the first three are the average BOLD signals from predefined seeds and the forth is the BOLD signal of a voxel. (**B**) 2D representation (mod) of the wavelet decomposition into time and frequency. (**C**) Coherence between the 4 time-series, obtained by Equation 1. (**D**) The three relative phases of the time-series for a certain time-frequency point. (**E**) These phase relations define the hierarchical order of the network. To infer directionality, an additional assumption is required, for example: positive Δ_*I,j*_ means the direction is *i*→*j*. (**F**) The directed pathway.

Figure 1 illustrates how the pathways are defined. A 4 time-series network is assigned a CP when its pathway is among the four relevant pathways included in the CP, as listed in Table 1.

To infer directionality within the CPs, i.e. whether a signal is progressing in a clockwise or counter-clockwise direction and to define its starting point (i.e. the directed pathway), an assumption or prior knowledge must be used. Note that in the definition of a CP only the phase-differences are considered and the functional connectivity amplitudes are not used. This distinguishes the current analysis of multivariate directed functional connectivity from other (bivariate) functional connectivity analyses, since the majority of other analyses focus on the amplitude. In other words, while most functional connectivity analyses are based on temporal similarity between pairs of time-series, our analysis is based on temporal hierarchy between multiple time-series. This means that in a hypothetical network of 4 identical time series, functional connectivity analyses that are based on temporal similarity (i.e. amplitude) will result with maximum functional connectivity, while our analysis that is based on temporal hierarchy will not assign a pathway to such a network since no temporal hierarchy can be defined. In this respect, our CPI measure is similar to the pairwise PLI (Phase Lag Index) measure. However, the key difference between our new method and the existing PLI method is that our method considers high order mutual information (characterized in terms of wavelet coefficients and phases) which makes it a multivariate measure versus the bivariate measure used in PLI. In other words, our method is a high order measure of directed functional connectivity versus the second order (pairwise PLI) measure of directed functional connectivity.

To identify significant CPIs in the whole brain, we constructed *3-seed-CPI*^*k*^*-SPMs*. A *3-seed-CPI*^*k*^*-SPM* is a statistical parametric map of CPI number k. *3-seed-CPI*^*k*^*-SPMs* are seed-like SPMs but instead of using a single seed, three seeds are used and a CPI based on 4 time-series networks is calculated. A *3-seed-CPI*^*k*^*-SPM* is composed of N CPI, where N is the number of voxels that were calculated using Equation 3. For each subject, the phases of 4 BOLD time-series were used in the CPI calculations. Each of the 4 time-series networks was constructed using 3 seed time-series and a fourth time-series of a voxel. These networks were calculated for every voxel and every subject. The 3 seed time-series were the average BOLD signals from 3 predefined regions, identified by masks based on Brodmann areas (see below).

We further used the 3 seed approach to identify a fourth seed that showed coupling with the initial (early visual) 3 seeds. This then allowed us to look at different combinations of 3 seeds and identify sets of four time-series within which we could identify significant circular patterns. This analysis was repeated for four frequency scales obtained by binning a wavelet decomposition into four cardinal frequency bins.

In summary, we used high order (multivariate) characterization of directed functional connectivity to construct statistical parametric maps (SPM) of nonlinear coupling between every voxel in the brain and a set of (three) seeds or reference regions.

## Results

### Circular patterns of the visual system

Resting-state fMRI data of 40 heathy young subjects were used to calculate *3-seed-CPI*^*k*^*-SPMs* of the ventral and the dorsal visual systems for the right and left hemispheres. Directed pathways from these networks were then inferred. Due to the interaction of the visual system with many other brain systems and our goal to characterize specifically the ventral and dorsal systems, three seeds were chosen in the early visual stages (V1, V2 and V3) and were confined to the dorsal or ventral systems. The fourth seed was chosen from the *3-seed-CPI*^*k*^*-SPMs* that were obtained using the first 3 seeds (for the ventral and dorsal systems, separately). All 4 combinations of *3-seed-CPI*^*k*^-*SPMs* calculations, each using 3 seeds, were performed. The 25 frequency scales of the wavelet analysis were grouped into 4 frequency scales: scale 1 = 0.01–0.03 Hz; scale 2 = 0.03–0.044 Hz; scale 3 = 0.047–0.067 Hz and scale 4 = 0.074–0.1 Hz.

#### The ventral system

For the ventral system, we used the following four predefined seeds: the primary visual cortex (in hereafter termed ‘V1’), the secondary visual cortex of the ventral system (in hereafter termed ‘V2_ventral’), the associative visual cortex of the ventral system (in hereafter termed ‘V3_ventral’) and a region that was found significant by *3-seed-CPI*^*k*^*-SPMs* of the first three seeds, and was within the fusiform gyrus (in hereafter termed ‘FG’). The analyses were done separately for seeds of the right and left hemispheres. A 3D plot of the four seeds is shown in Supplementary Figure 1. 3*-seed-CPI*^*k*^*-SPMs* were calculated for the following seed combinations: (1) R1 = V1, R2 = V2_ventral, R3 = V3_ventral; (2) R1 = V1, R2 = V2_ventral, R3 = FG; (3) R1 = V1, R2 = V3_ventral, R3 = FG and (4) R1 = V2_ventral, R2 = V3_ventral, R3 = FG.

**Figure 2:**
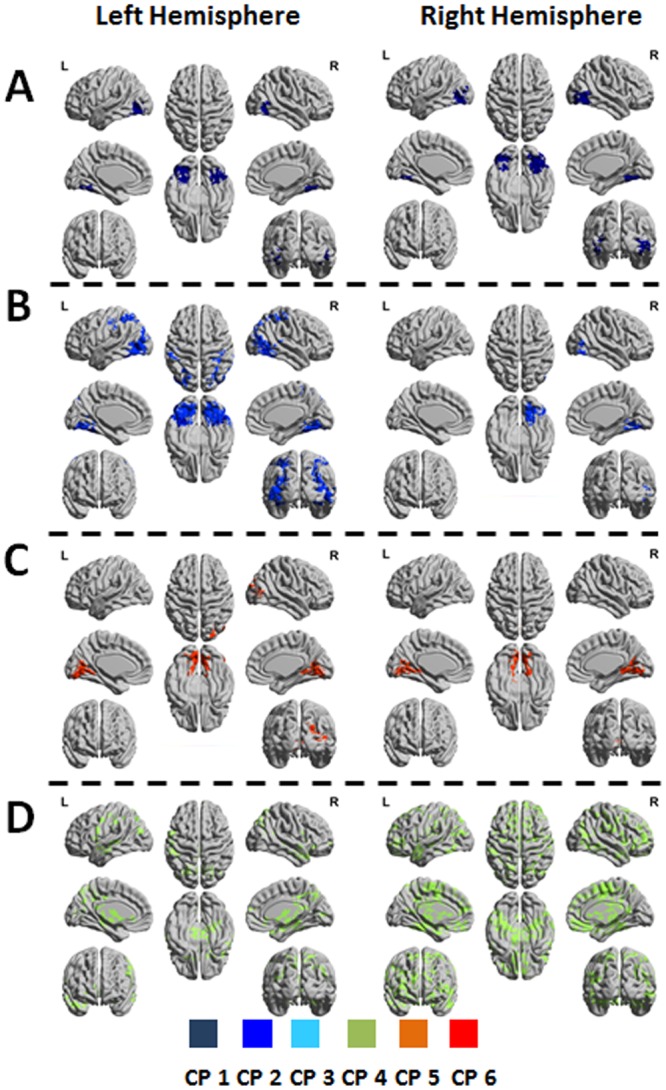
3-*seed-CPI*^k^*-SPMs* of the ventral stream. The figure shows 3D representations of significant clusters included in the circular-patterns (CPs). Different CPs are indicated by the color bar. (**A**) *3-seed-CPI*^k^*-SPMs* for seed combination 1 (R1 = V1, R2 = V2_ventral, R3 = V3_ventral) of the left and right hemispheres. (**B**) *3-seed-CPI*^k^*-SPMs* for seed combination 2 (R1 = V1, R2 = V2_ventral, R3 = FG) of the left and right hemispheres. (**C**) *3-seed-CPI*^k^*-SPMs* for seed combination 3 (R1 = V1, R2 = V3_ventral, R3 = FG) of the left and right hemispheres. (**D**) *3-seed-CPI*^k^*-SPMs* for seed combination 4 (R1 = R2_ventral, R2 = V3_ventral, R3 = FG) of the left and right hemispheres. ‘FG’ indicates a cluster defined within the fusiform gyrus.

For the first seed combination, all significant networks obtained by the *3-seed-CPI*^*k*^*-SPMs* calculations were characterized by CP no. 1 and were found in frequency scales 2 and 3, for both hemispheres. A ‘network’ refers hereinafter to the functional connectivity of 4 time-series; the BOLD signals of the three seeds and a voxel, defined by Equation 2 that passed the statistical cutoff (see method). In this respect, the number of networks equals to the number of significant voxels. These voxels are aggregated in clusters due to the applied cluster-size statistical threshold and are shown by 3D plots. Figure 2A show 3D plots of the 3*-seed-CPI*^*k*^*-SPMs* networks for seeds in the left hemisphere (left column) and seeds in the right hemisphere (right column). All significant clusters were located in the fusiform gyrus, known to be part of the ventral stream. Table 2 presents the number of significant networks, their CP and frequency scale.

**Table 2:** Numbers of ‘4 time series’ networks of the ventral and the dorsal systems.

		Left Hemisphere				Right Hemisphere			
		Scale 1	Scale 2	Scale 3	Scale 4	Scale 1	Scale 2	Scale 3	Scale 4
**Ventral System**									
Combination 1	CP 1		3982	1144			3997	3834	
Combination 2	CP 1		2325						
	CP 2	12384	6098			1331	1057		
Combination 3	CP 6		1924	4034			2424	1799	
Combination 4	CP 4	15993				46988			
**Dorsal System**									
Combination 1	CP 4	30225	31464		2667	1173	15832		
Combination 2	CP 3	1665	2530			1037	2989		
	CP 2							3424	
	CP 4	8706					9304		
Combination 3	CP 5	4665	1746				1552		
	CP 4	59793	40520	18741		3595	29119	8955	
Combination 4	CP 4	95183	35675	81227	1568	9276	29171	40993	

Networks were made from time series of 3 seeds and a voxel and are defined by the *3-seed-CPI*^k^*-SPMs*. Numbers of networks are given for the four seed combinations arranged by their CP’s, frequency scales and hemisphere. For the ventral system the seed combinations were: combination 1: V1, V2_ventral, V3_ventral; combination 2:V1, V2_ventral, FG; combination 3: V1, V3_ventral, FG; combination 4: V2_ventral, V3_ventral_FG. For the dorsal system the combinations were: combination 1: V1, V2_dorsal, V3_dorsal; combination 2:V1, V2_dorsal, SP; combination 3: V1, V3_dorsal, SP; combination 4: V2_dorsal, V3_dorsal_SP. ‘FG’ indicates a cluster within the fusiform gyrus and ‘SP’ indicates a cluster defined within the superior parietal gyrus.

For the second seed combination, significant networks obtained by the *3-seed-CPI*^*k*^*-SPMs* calculations for the left hemisphere were characterized by CP no. 1 in scale 2 and CP no. 2 in scales 1 and 2. For the right hemisphere, significant networks were characterized by CP no. 2 in scales 1 and 2 (Table 2). Figure 2B show 3D plots of the 3*-seed-CPI*^*k*^*-SPMs* networks for seeds in the left hemisphere (left column) and right hemisphere (right column). Significant clusters were found within the ventral system including V2_ventral, V3_ventral and the fusiform gyrus. Significant clusters were also found in the dorsal system including V3_dorsal and regions within the temporal gyrus.

For the third seed combination, significant networks obtained by the *3-seed-CPI*^*k*^*-SPMs* calculations for the left hemisphere were characterized by CP no. 6 in scales 2 and 3. For the right hemisphere, significant networks were characterized by CP no. 6 in scales 2 and 3 (Table 2). Fig. 2C show 3D plots of these 3*-seed-CPI*^*k*^*-SPMs* networks for the left and right hemispheres. Significant clusters were found bilaterally in the extrastriate cortex, mainly within its ventral parts.

For the fourth seed combination, significant networks obtained by the *3-seed-CPI*^*k*^*-SPMs* calculations for the left hemisphere were characterized by CP no. 4 in scale 1. For the right hemisphere, significant networks were characterized by CP no. 4 in scale 1 (Table 2). Figure 2D show 3D plots of the 3*-seed-CPI*^*k*^*-SPMs* for seeds in the left hemisphere (left column) and right hemisphere (right column). Whereas significant clusters of the left hemisphere were mainly restricted to associative somatosensory and frontal areas, the right hemisphere included multiple clusters distributed across the cortex and subcortex.

#### The dorsal system

For the dorsal system, we used the following four predefined seeds: the primary visual cortex (V1), the secondary visual cortex of the dorsal system (in hereafter termed ‘V2_dorsal’), the associative visual cortex of the dorsal system (in hereafter termed ‘V3_dorsal’) and a fourth seed that was constructed by the use of significant clusters obtained by *3-seed-CPI*^*k*^*-SPM*s of the first three seeds and included clusters within the superior parietal gyrus (in hereafter termed ‘SP’, see method). The analyses were done for seeds of the right hemisphere and for seeds of the left hemisphere separately. A 3D plot of the four seeds is shown in Supplementary Figure 1. 3*-seed-CPI*^*k*^*-SPMs* were calculated for all combinations of choosing three out of the four seeds: (1) R1 = V1, R2 = V2_dorsal, R3 = V3_dorsal; (2) R1 = V1, R2 = V2_dorsal, R3 = SP; (3) R1 = V1, R2 = V3_dorsal, R3 = SP and (4) R1 = V2_dorsal, R2 = V3_dorsal, R3 = SP.

**Figure 3:**
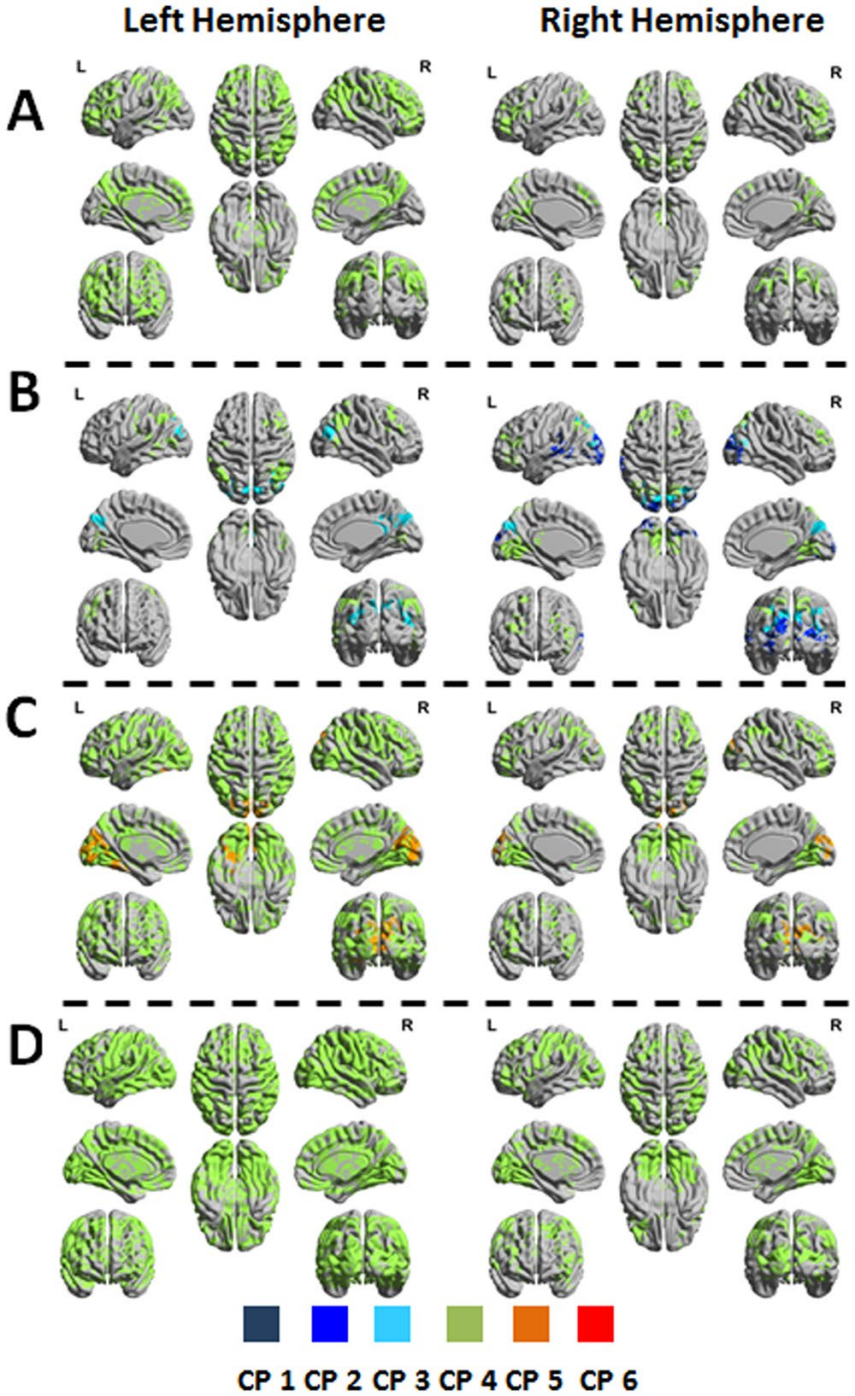
3-*seed-CPI*^k^*-SPMs* of the dorsal stream. The figure shows 3D representations of significant clusters included in the circular-patterns (CPs). Different CPs are indicated by the color bar. (**A**) *3-seed-CPI*^k^*-SPMs* for seed combination 1 (R1 = V1, R2 = V2_dorsal, R3 = V3_dorsal) of the left and right hemispheres. (**B**) *3-seed-CPI*^k^*-SPMs* s for seed combination 2 (R1 = V1, R2 = V2_dorsal, R3 = SP) of the left and right hemispheres. (**C**) *3-seed-CPI*^k^*-SPMs* for seed combination 3 (R1 = V1, R2 = V3_dorsal, R3 = SP) of the left and right hemispheres. (**D**) *3-seed-CPI*^k^*-SPMs* for seed combination 4 (R1 = R2_dorsal, R2 = V3_dorsal, R3 = SP) of the left and right hemispheres. ‘SP’ indicates a cluster defined within the superior parietal gyrus.

For the first seed combination, all significant networks obtained by the *3-seed-CPI*^*k*^*-SPMs* calculations were characterized by CP number 4. For the left hemisphere, networks were found in scales 1,2,4 and for the right hemisphere in scales 1 and 2 (Table 2). Fig. 3A show 3D plots of the 3*-seed-CPI*^*k*^*-SPMs* for seeds in the left hemisphere (left column) and seeds in the right hemisphere (right column). Networks in the right hemisphere were found within the extrastriate and associative somatosensory cortices and in addition in lateral frontal areas, including the frontal eye fields (FEF). Networks of the left hemisphere were found in the same areas with larger clusters. For both hemispheres, the distribution of clusters highly overlapped with the dorsal attention network (DAN), including the intraparietal sulcus (IPS) and the FEF. For the left hemisphere, additional clusters were indicated in the ventral attention network (VAN), including the temporoparietal junction (TPJ) and ventral frontal cortex (VFC).

For the second seed combination, significant networks obtained by the *3-seed-CPI*^*k*^*-SPMs* calculations for the left hemisphere were characterized by CP no. 3 in scales 1 and 2 and CP no. 4 in scale 1. For the right hemisphere, significant networks were characterized by CP no. 2 in scale 3, CP no. 3 in scales 1,2 and CP no. 4 in scale 2 (Table 2). Fig. 3B show 3D plots of the 3*-seed-CPI*^*k*^*-SPMs* for seeds in the left hemisphere (left column) and seeds in the right hemisphere (right column). Significant clusters of CP numbers 2 and 3 were within the extrastriate and associative somatosensory cortices, which are known to be a part of the dorsal system, whereas significant clusters of CP no. 4 included also remote regions such as the frontal gyrus and specifically the FEF. The left TPJ, IPS and FEF were included in networks of both left and right hemispheres.

For the third seed combination, significant networks obtained by the *3-seed-CPI*^*k*^*-SPMs* calculations for the left hemisphere were characterized by CP no. 4 in scales 1,2,3 and CP no. 5 in scales 1,2. For the right hemisphere, significant networks were characterized by CP no. 4 in scales 1,2,3 and CP. no 5 in scale 2 (Table 2). Fig. 3C show 3D plots of the 3*-seed-CPI*^*k*^*-SPMs*. Significant clusters of CP no. 5 of the left hemisphere were found within the striate and extrastriate cortices, and significant clusters of CP no. 4 for both hemispheres were distributed in the entire dorsal system, including regions in the DAN and VAN. Note that much more networks were found for seeds in the left hemisphere compared to the right hemisphere, with widespread involvement of almost the entire cortex.

For the fourth seed combination, all significant networks were characterized by CP no. 4. For the left hemisphere, networks were found in scales 1,2,3 and 4. For the right hemisphere, networks were found in scales 1,2 and 3 (Table 2). Figure 3D show 3D plots of the 3*-seed-CPI*^*k*^*-SPMs*. Significant clusters were found within the ventral system and the entire bilateral dorsal system. Whereas networks of seeds in the left hemisphere covered almost the entire cortex and subcortex, networks of the right hemisphere were smaller, less distributed and included the DAN.

Table 2 suggests that there was no effect of frequency (scale). Permutation-based non-parametric tests show that for the dorsal system the number of networks for seeds in the left hemisphere was significantly higher than the number of networks for seeds in the right hemisphere in seed combinations 3 and 4. This was due to more networks in the left hemisphere that connected the dorsal system with non-visual regions characterized by CP no. 4 (e.g. the DAN). In contrast, the number of networks that were confined to the dorsal system, were about equal between the hemispheres. An additional finding of greater significance was the higher number of networks in the dorsal system compared to the ventral system, for either right or left hemispheres. These results support pathways of information flow specifically between the dorsal visual system and the VAN and DAN.

### Comparison of 4 time-series to pairwise coherences analyses

To examine the extent to which the results above are unique for the new multivariate 4 time-series analysis, we compared it to bivariate pairwise coherence analyse. Specifically, we aimed to test whether the differences found between the dorsal and ventral systems namely, a greater extent of connectivity of the left dorsal system to non-visual regions, can be also identified by second order pairwise coherence analysis.

Pairwise functional connectivity was obtained similarly to previous studies^6,24,25^:

4 $$F{C}_{seed,j}(\omega ,t)=({W}_{\omega ,t}^{seed})\cdot {({W}_{\omega ,t}^{j})}^{\ast }={A}_{seed,j}(\omega ,t)\cdot \exp (i{\theta }_{seed,j}(\omega ,t))$$

Average over time was performed for the resting-state data as in the multivariate analysis. For testing coherence phase significance, we used the Phase Lag Index (PLI)^22,23^ method since it is more similar to the CPI than the phase locking value (PLV). The V2 region was chosen for comparison, and the pairwise coherence analyses was computed for the V2_ventral and V2_dorsal seeds of the left and right hemispheres, the same seeds that were used in the multivariate analysis. *Seed-PLI-SPMs* were obtained by calculating pairwise coherences (between a seed and a voxel time-series, for all voxels in the brain) for each frequency, using wavelet analysis with the same frequency scales. *Seed-PLI-SPMs* of V2_ventral and V2_dorsal seeds of the right and left hemispheres are shown in Supplementary Figure 2.

*Seed-PLI-SPMs* of different frequency scales were largely overlapping, suggesting no effect of scale, similarly to the finding for the *3-seed-CPI*^*k*^*-SPMs*. As seen in Supplementary Figure 2, *Seed-PLI-SPMs* of V2 regions were confined to the early visual areas and were almost invariant to left and right choices. This contrasts with the results for the *3-seed-CPI*^*k*^*-SPMs* that were very different for the left and right hemispheres (Figs 2 and 3). *Seed-PLI-SPMs* of V2_ventral had about twice significant pairwise coherences compared with the V2_dorsal which is opposite to the results obtained with *3-seed-CPI*^*k*^*-SPMs*. Note also that the number of significant pairwise coherences was much lower compared with the number of significant networks obtained by the multivariate analysis.

To test whether the differences found in coherence phases between the multivariate and bivariate analyses were complimented by differences in coherence strength (amplitude), we calculated the average coherence strength for both analyses over all voxels. Namely, the average bivariate coherence strength of the left and right V2_ventral and V2_dorsal seeds over all voxels and frequency scales. Similarly, we calculated the average multivariate coherence strength of the left and right ventral and dorsal 3-seed networks of combination IV (using R1 = V2_ventral, R2 = V3_ventral, R3 = FG for the ventral stream and R1 = V2_ventral, R2 = V3_dorsal, R3 = SP for the dorsal stream), over all voxels. Supplementary Figure 3 shows these results. Whereas there were no differences between ventral and dorsal bivariate coherence strengths for all scales, significant differences were found between the ventral and the dorsal 4 time-series networks’ strength for the right and the left hemispheres (with p < 0.01 paired t-test for all comparisons besides the comparison of the left hemisphere at scale 1). For the 4-timeseries analysis, the ventral stream had stronger coherence strength for all scales with differences in strength a function of scale. Note that the amplitude values, for the bivariate and the multivariate analyses, changed approximately as 1/f, as expected.

Taken together, the results suggest marked differences between the 4 time-series and pairwise coherence analyses with higher specificity of the former.

### Directed pathways in the visual system

To obtain directed pathways from the CPs, two assumptions are needed. A global assumption for the directionality of signal progression within the CP and a local assumption for the region in which the pathway starts. We assume that fMRI BOLD time-lags are related to neuronal time-lags and therefore BOLD signals, in the majority of pathways, progress from lower to higher visual areas. This is based on previous studies suggesting neuronal flow from V1 to higher visual areas^12,13^. To determine the global flow directionality, we used the following two considerations: one, in all the four possible seed combinations, the *3-seed-CPI*^*k*^*-SPMs* calculations were performed with the first three seeds (R1, R2 and R3 in Table 1) chosen according to the expected hierarchy of the system, where R1 is the lower visual area of the three seeds and R3 the highest visual area (e.g. R1 = V1 R2 = V2 and R3 = V3). Second, as shown in Figs 2 and 3 and Table 2, most networks were characterized by CP no. 4 (i.e. R4-R3-R2-R1). Taking together the above and expecting that the majority of pathways will correspond to signal flow from lower to higher visual areas, this implies that the directionality within CP no. 4 is R1→R2→R3→R4 which is a counter-clockwise directionality. Although Equations 1 and 2 do not infer that the same directionality exists in all networks (i.e. networks with different seeds), we argue that once directionality is defined it is valid to all CPs. This is since BOLD temporal signals of all regions are locked such that the phase-difference’s signs, that determine directionality, are related. Consequently, to construct directed pathways for all other CPs, we need also to assume counter-clockwise directionality. For the local definition of where the pathway starts in each CP, the expected low ↔ high visual hierarchal organization is used. Note, that even without using the last assumption, our analysis infer the existence of both feedforward and feedback pathways in the visual system.

#### The ventral system

Four CPs were identified in the ventral system. For the first seed combination, CP no. 1 (V1-V2-V3-R4) was identified where R4 included clusters within the fusiform gyrus. Counter clockwise directionality and low ↔ high hierarchal organization suggest the directed pathway: FG → V3 → V2 → V1. For the second seed combination, CP no. 2 (V1-V2-R4-FG) was identified where R4 included clusters within V2_ventral, V3_ventral, the fusiform gyrus and regions known to be part of the left dorsal stream (e.g. the middle temporal gyrus). Counter clockwise directionality and low ↔ high hierarchal organization suggest the following directed pathways: FG → V3/V2 → V2 → V1 and FG → MT → V2 → V1. For the third combination, CP no. 6 (V1-R4-V3-FG) was identified where R4 included clusters within V2_ventral. Counter clockwise directionality and low ↔ high hierarchal organization suggest the directed pathway: FG → V3 → V2 → V1. For the fourth seed combination, CP no. 4 (V2-R4-FG-V3) was identified where R4 included various distributed clusters in both hemispheres. Applying the same assumptions as above, this leads to the directed pathway: V2 → V3 → FG → R4.

#### The dorsal system

Seven CPs were identified in the dorsal system. The majority of networks were characterized by CP no. 4 (96%). For the first seed combination, CP no. 4 (V1-R4-V3-V2) was identified where R4 included largely distributed clusters. Counter clockwise directionality and low ↔ high hierarchal organization suggest the following directed pathway: V1 → V2 → V3 → R4. For the second seed combination, CPs no. 2,3, and 4 were identified. R4 clusters in CP no. 2 (V1-V2-R4-SP) were mainly found within the extrastriate cortex. Counter clockwise directionality and low ↔ high hierarchal organization suggest the directed pathway: V1 → V2 → V2/V3 → SP. R4 in CP no 3 (V1-SP-R4-V2) included clusters within the superior parietal gyrus. Counter clockwise directionality and low ↔ high hierarchal organization suggest the directed pathway: V1 → V2 → R4 → SP. CP no. 4 (V1-R4-SP-V2) included R4 clusters in multiple remote areas of the dorsal stream. Counter clockwise directionality and low ↔ high hierarchal organization suggest the pathway: V1 → V2 → SP → R4. For the third seed combination, CPs no. 4 and 5 were identified. CP no. 4 (V1-R4-SP-V3) included R4 clusters across the entire dorsal stream. For this CP, counter clockwise directionality and low ↔ high hierarchal organization suggest the pathway: V1 → V3 → SP → R4. CP no. 5 (V1-SP-V3-R4) included R4 clusters within the striate and extrastriate cortices, and its directionality is suggested to be: V1 → R4 → V3 → SP. For the fourth seed combination, CP no. 4 (V2-R4-SP-V3) was identified where R4 clusters were mainly distributed outside the visual system, in addition to clusters within the entire dorsal and ventral streams. Counter clockwise directionality and low ↔ high hierarchal organization suggest the pathway: V2 → V3 → SP → R4.

**Figure 4:**
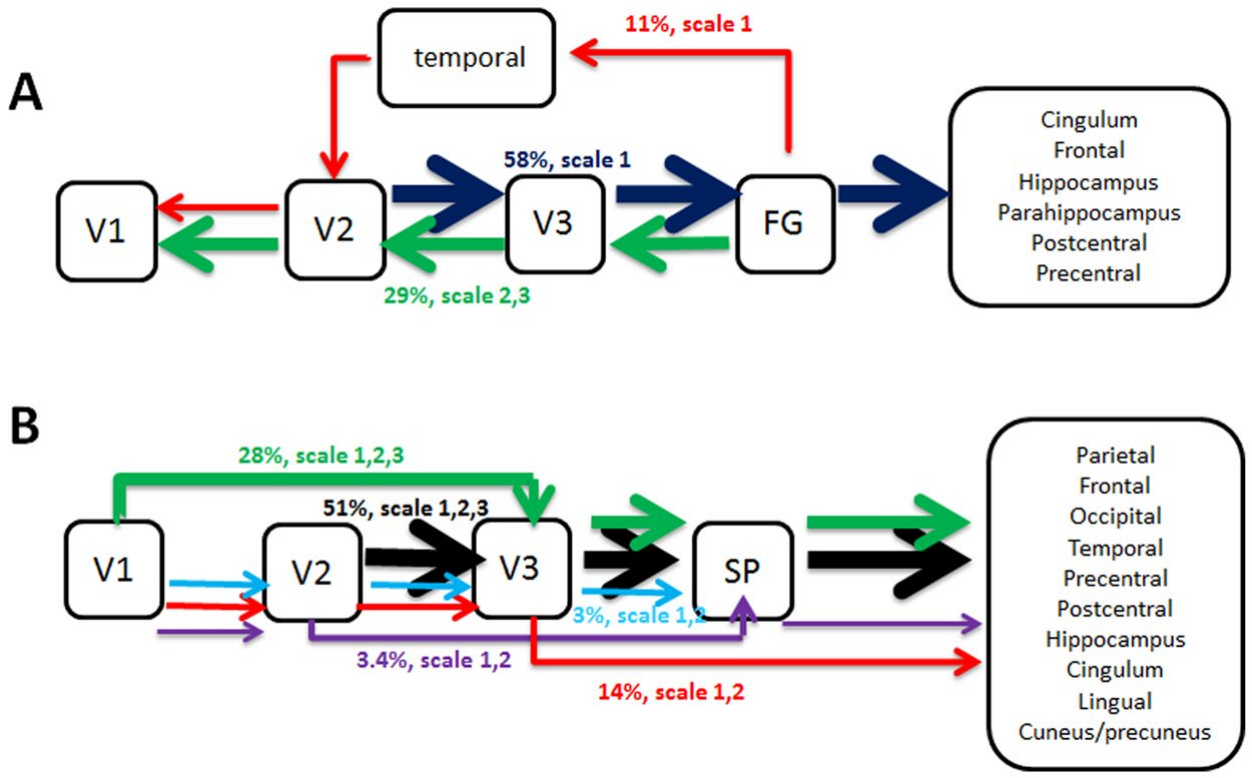
Directed functional pathways of the ventral and dorsal systems during resting-state acquisition with eyes open. Pathways were inferred from the circular-patterns (CPs) that were obtained by the 3*-seed-CPI*^*k*^*-SPMs* calculations. The method enables to infer directed functional pathways of 4 stages. Solid arrows indicate the inferred directions and different pathways are indicated by colors. The percentage of each pathway and its frequency scale are denoted above each arrow, and the widths of the arrows are proportional to the percentage of the pathway. (**A**) Directed pathways of the ventral system. (**B**) Directed pathways of the dorsal system.

Figure 4 summarizes these pathways for the ventral (4A) and dorsal (4B) systems in a box-arrow diagram. In the figure, arrows represent the predominantly direction of coupling. For both streams, pathways that connect the visual system with other systems (e.g. frontal regions) were feedforward. Pathways within the early visual cortex were of opposite directionality between the streams: dorsal pathways were feedforward while ventral pathways were feedback. Note, that whereas pathways of the ventral stream that included regions outside the visual system didn’t contain the striate cortex, pathways of the dorsal stream that included regions outside the visual system involved the striate and the extrastriate cortices.

## Discussion

Using a novel high order multivariate functional connectivity analysis method that is based on nonlinear coherence with resting-state fMRI data of 40 healthy subjects, we have identified patterns of signal progression in the ventral and dorsal visual systems, with no need for any assumption. The analysis enables to obtain functional patterns of signal progression in a network of 4 time-series In our approach, three are the average BOLD signals of predefined regions and the forth is the BOLD temporal signal of a voxel. A circular-pattern flow, one out of possible 6 which characterizes the signal progression, was assigned to each network using only the network’s phases (Fig. 1). In this respect, our analysis is different from most other connectivity analyses which utilize connectivity strength. An intrinsic limitation of the analysis is that it is limited to four network nodes. This may possibly result with an over simplified description of signal progression and with a limited ability to characterize accurately a certain pathway. However, this remains an advantage of the method over existing functional connectivity analyses that are limited to two nodes. Another limitation that is specific for resting-state data is the need to average along time. The wavelet coefficients used in the analysis were associated with time-averaged signals and thus the directionality found is the average directionality. This limitation, however, is common to all resting-state data analyses. We note that the analysis can be used with other imaging types including stimulus driven data for which no time averaging is required enabling dynamic study. Last, for simplicity, we have limited our analysis to continuous pathways. The case of general flow patterns will be dealt in a future study.

Our results were obtained with resting-state fMRI data therefore, could be limited to this type of data. However, increasing evidence suggests that neurons *in vivo* are active in the absent of external input and that this spontaneous activity has a coherent structure. In support of this possibility, neurophysiological studies have shown that the activity of individual neurons is similar across spontaneous and stimulus-driven brain states^26,27^. Furthermore, multiple neuroimaging studies have shown a correspondence between spontaneous and stimulus-driven states consistent with the idea that the brain has an intrinsic functional architecture^28,29^. Additionally, interhemispheric coupling of somatosensory regions and of auditory cortices were synchronized in response to stimulus at 20–30 Hz and 7–12 Hz respectively and these frequencies were also selective to these interregional interactions during spontaneous activity^30^. Consequently, we assume that our findings are applicable to the visual system in general, and in the discussion below we refer to previous findings of single- or multi-unit recordings as well as of stimulus driven data to support our findings.

Coherence between 4 time-series at a given time-frequency window implies that all four time-series are mutually coupled. This means that key characteristics of the signals (within these windows) coexist in all the time-series. For such mutual coupling to occur, interaction between the four time-series must happen simultaneously (i.e., within the time-resolution of the data). This implies that the time resolution of data acquisition might affect the results, giving a higher weight to faster signal transfer processes. Several other scenarios that enable such mutual coherences can be considered, including recurrent connections. The issue of whether we can discern feedforward (or unilateral feedback) connections from reciprocal or recurrent connections is outstanding. Local processing and recurrent connections could either increase or decrease phase differences, with subsequent effects on our circular pattern statistics. We therefore limit ourselves to interpreting any significant hierarchical ordering of phase delays in terms of predominantly unidirectional (feedforward or feedback) coupling. We will present a simulation analysis of the effect of recurrent connections in a subsequent work.

By obtaining *3-seed-CPI*^*k*^*-SPMs* and *seed-PLI-SPMs* using the same seeds of the V2 region and equal statistical thresholds, we showed that the clusters found to be connected to the V2 seeds by pairwise coherences were limited to the early visual system, were largely overlapped for the right and left hemispheres and were smaller compared to those detected by the 4 time-series analysis (Supplementary Figure 2). In contrast, major differences between the ventral and dorsal systems and between the hemispheres were found using the 4 time-series multivariate analysis (Figs 2 and 3). Furthermore, the average bivariate pairwise coherences’ strengths over all voxels of the V2_dorsal and V2_ventral seeds were found to be equal for all scales (Supplementary Figure 3). In contrast, the average multivariate 4 time-series coherence’s strengths over all voxels for networks of seed combination IV (and the other seed combinations) were found to be different between the ventral and the dorsal systems (Supplementary Figure 3). The dorsal system was shown to have lower coherence’s strength and the differences in strength were scale depended. The fact that such differences were only detected by the new analysis, suggests that it can distinguish between diverse coherence types that are not detectable by pairwise coherences. With regards to the reasons for dorsal coherence’s strength to be lower compared to ventral coherence’s strength, we note that in general coherence’s strength decreases as the phase-differences (between the time-series) increase. This is also evident from the observed scale dependency shown in Supplementary Figure 3: Assuming a fixed or pseudo-fixed time-delay between time-series for the different scales, the corresponding phase-differences increase with scale. It remains to be explained why dorsal networks had larger phase-differences. At this level we can only speculate that the higher recurrent flow that is expected in the dorsal system resulted with an effective higher phase-differences. This issue will be further studied in the future.

One of the most pronounced finding was the higher number of networks for the dorsal system compared to the ventral system, where most of these were networks that connected visual and non-visual systems. This suggests that signal transfer between visual and non-visual regions has different characteristics in the ventral and dorsal systems. Indeed, multiple studies have shown faster signal flow in the dorsal compared to the ventral system^18^. This fast flow in the dorsal system (the’magnocellular advantage’) was suggested to enable top-down influence of higher brain regions on visual processing and organization by multiple local and remote recurrent loops. We therefore suggest that the higher number of networks observed in the dorsal system is the result of faster neuronal flow in that system.

The number of networks in the left dorsal system for seed combinations 3 and 4 was found to be higher than in the right hemisphere. In these seed combinations, the last two seeds were V3_dorsal and a seed within the parietal cortex (the SP seed). The parietal cortex in the non-dominant hemisphere (right hemisphere of 77.5% of the subjects) is well known to be involved in visualization of spatial relationships and imagery as well as non-verbal memory. An injury to the parietal cortex in the non-dominant hemisphere might cause various “visual-related” clinical symptoms such as spatial disorientation, constructional apraxia including difficulties in dressing, drawing and navigating, loss of imagery and neglect of the left-side world including space and self. No such symptoms are related to parietal injuries in the dominant hemisphere (left hemisphere) where injury leads to non-visual symptoms such as Gerstmann syndrome, receptive aphasia and mathematical problems. Consequently, the right parietal lobe is thought to be more involved with visual processing. Although the intuitive assumption might be that the right hemisphere should have more visual networks, our data shows the opposite. Based on our findings, we claim that the more local and less distributed connectivity found in the right hemisphere compared to the left, implies higher sensitivity to visual deficits following injuries and therefore relates to the clinical symptoms described above. In other words, we suggest that the higher number of visual networks for the left hemispheres and their unspecific widespread distribution with multiple clusters that almost covered the whole cortex might reduce its sensitivity for visual deficits following injuries. Furthermore, the data suggests that these networks are feedforward pathways that include V3 and the parietal cortex, suggesting a strong dependency on hierarchical order (i.e. including all visual stages).

Our results showed that the dorsal and ventral systems are functionally connected. Networks connecting the dorsal to the ventral systems were mainly of the left hemisphere with the connecting region in the superior parietal gyrus (seed 4). This is in line with several studies suggesting that information retro-injected from the parietal cortex is used to guide further processing of parvocellular and koniocellular information in the inferotemporal cortex^17^. Much fewer networks were found to connect the ventral system to the dorsal system, suggesting that interaction between the two systems is predominantly from the dorsal system.

Figure 4 presents the predominant direction of coupling found during resting-state acquisition with eyes open. As seen, although the majority of pathways in both streams were feedforward, major differences were found between the two streams. The directionality of the pathways shown in Fig. 4 is the direct consequence of the assumption of lower to higher processing and of low ↔ high hierarchal organization. However, the use of other assumptions (e.g. opposite directionality) will retain the major differences found between the dorsal and ventral systems. Within the assumption of lower to higher processing, almost all feedforward pathways of the dorsal system were connected to non-visual systems and initiated in the early visual stages (V1 and V2). In contrary, about half of the pathways in the ventral system were feedforward, all connected to non-visual systems and initiated in V2. Furthermore, all pathways that were confined to the striate and extrastriate cortices in the ventral system were feedback pathways that initiated in the fusiform gyrus while the few that were confined to these regions in the dorsal system were feedforward and initiated in V1. These findings demonstrate a major organization difference between the two streams.

## Conclusions

We extended our novel multivariate analysis method to obtain directed functional pathways of BOLD signal progression within the brain. In contrast to most other connectivity analyses that are based on temporal similarity (i.e. connectivity strength), our analysis is based on the temporal order between multiple regions (i.e. coherences). We show that the new analysis contains more information compared with functional connectivity based on pairwise coherences since it discriminates between the two streams. Within the limitation of interpreting hierarchical ordering by means of phase delays in terms of unidirectional (feedforward or feedback) coupling, we show that the organizations of the ventral and dorsal systems are remarkably different. Whereas the dorsal system consisted of mainly feedforward pathways that included the striate, extrastriate cortices and non-visual regions, the ventral system included fewer pathways that were classified into two types: feedback pathways that initiated in the fusiform gyrus and either confined to the striate and extrastriate cortices or connected to the dorsal system, and feedforward pathways that initiated at V2 and included non-visual regions. Finally we note that the new analysis can be further extended to include more regions, applied on other neuroimaging data types (e.g. MEG) and thus open possibilities for characterization of complex neural systems.

## Method

### Subjects

The same sample was used as in our previous publication^6^. The sample was composed of 42 healthy control young subjects (20 women, age: 24.14 ± 2.67 years). Subjects were recruited among students at the Hebrew University of Jerusalem. Before inclusion, all subjects were clinically interviewed using Structured Clinical Interview for DSM-IV (SCID-CV) to exclude past or present psychiatric or neurological disorders. One male subject was excluded due to family history of schizophrenia and another male subject was excluded due to anxiety during the MRI scan, which yielded a final sample of 40 subjects (20 women, age: 24.15 ± 2.74 years). 13 men and 18 women were right handed. The study was approved by the Hadassah Hebrew University Medical Center Ethics Committee. All participants provided written informed consent prior to inclusion in the study in compliance with the Declaration of Helsinki.

### MRI data acquisition

Magnetic resonance images were acquired with a 3T Siemens MR scanner at the Neuroimaging Unit of the Edmond and Lily Safra Center for Brain Sciences at the Hebrew University of Jerusalem. Each participant underwent 10-minute of resting-state functional MRI during which they were instructed to fixate on a visual crosshair, remain still and awake. Immediately after the scan, each participant confirmed not falling asleep. Functional images were acquired using T_2_^*^-weighted gradient-echo echo-planar imaging (GE-EPI) sequence with TR = 2 sec, TE = 30 ms, image matrix = 64 × 64, field of view = 192 × 192mm, flip angle = 90°, resolution = 3 × 3 × 3mm, interstice gap = 0.45 mm. Each brain volume comprised 30 axial slices, and each functional run contained 300 image volumes. High resolution anatomical images were acquired using a sagittal T1-weighted magnetization-prepared rapid acquisition gradient echo (MP-RAGE) sequence with TR = 2300 ms, TE = 2.98 ms, inversion time = 900 ms, flip angle = 9°, resolution = 1x1x1mm. T1-weighted images were acquired for coregistration and normalization of the functional images.

### Functional MRI data preprocessing and functional connectivity analysis

Standard initial preprocessing of functional MRI data was done using Statistical Parametric Mapping (SPM8, Wellcome Trust Centre for Neuroimaging, London, United Kingdom, http://www.fil.ion.ucl.ac.uk/spm/software/spm8). First, functional images were spatially realigned using a least squares approach and a six parameter (rigid body) spatial transformation. Subsequently, functional images were coregistered to high resolution T1 anatomical images, normalized to Montreal Neurological Institute (MNI) space and resampled at an isotropic voxel size of 2 mm. The normalized images were smoothed with an isotropic 8 mm full-width-at-half-maximum Gaussian kernel. Motion parameter estimates were carefully checked for each individual separately. Subjects were excluded if head motion reached voxel size in any direction. Average maximal displacement for subjects was <1 mm. Further preprocessing was done using CONN toolbox^31^. Censoring was done according to the method of Power *et al*.^32^. Confounds were removed by regression, including the six motion parameters, their first order derivatives, scrubbing parameters and 3 principle components of the CSF and the white matter. Prior to regression of principal components, the white matter and CSF masks were eroded to ensure that only pure white matter or CSF signal was regressed from the data. Potential effects of scan initiation were removed by applying a step function convolved with the hemodynamic response function. Regression-out of confounds was done to minimize effects of motion and potential physiological and non-neuronal signals such as cardiac and respiratory signals, without the risk of artificially introducing anticorrelations into the functional connectivity estimates^33–37^. Last, linear detrending and band-pass filtering (0.009-0.08 Hz) were applied. Note that a narrower frequency range was used compared to our previous publication, since previously most networks in the visual system were found in the lowest frequency range.

### Multiple-region directed connectivity analysis

All further calculations were performed with IDL version 8.2.0 (Exelis Visual Information Solutions, Inc.) using custom-developed software. The complex Morlet wavelet functions were chosen for wavelet analysis since they have been shown to provide a good trade-off between time and frequency localization^38^. We used 3 for the smallest scale, 2 for the time resolution and 25 scales to cover the entire frequency window. Wavelet software was provided by C. Torrence and G. Compo available at: http://paos.colorado.edu/research/wavelets ^39^. The 25 frequency scales were further averaged into 4 frequency scales: 0.01–0.03 Hz; 0.03–0.044 Hz; 0.047–0.067 Hz and 0.074–0.1 Hz.

### Selection of brain regions

The mask of Brodmann area 17 (primary visual area) obtained from the Talairach Daemon atlas^40^ was manually divided to left and right hemispheres using MRIcron toolbox^41^. The masks of Brodmann areas 18 (secondary visual area) and 19 (associative visual area) were manually divided to left, right, ventral and dorsal parts using the same toolbox. This resulted with a total of 10 masks: left and the right V1, left and right ventral V2, left and right dorsal V2, left and right ventral V3 and left and right dorsal V3. These masks were visually inspected to match the corresponding areas and no overlap was confirmed. Masks for the fourth seeds (for the ventral and dorsal systems) were obtained from the *3-seed-CPI*^*k*^*-SPMs* obtained by the first three seeds, to ensure their inclusion in the systems of interest. Brodmann masks rather than individualized ROIs were used to simplify the experiments and analysis and to minimize differences in seeds’s volumes between individuals.

### Statistical analysis

#### CPI null distributions

To define the significance of the Circular-Pattern-Indexes (CPI), permutation tests were used and the CPI’s null distributions were calculated for each CP and each scale. The null distributions were calculated as following: 4 time-series networks were constructed by 4 BOLD signals that were the average BOLD signals from different visual Automated Anatomical Labeling (AAL)^42^ regions, where each signal was taken from a different subject. These seeds where chosen from the visual system to obtain null distributions close as possible to the data. No randomization was used on the choice of the seeds. By using time-series from different subjects we guaranteed that no correlation between them is expected. For each network, functional connectivity (Equation 1) was calculated for each frequency separately and the phases were obtained according to Equation 2. Averaging of the wavelet coefficients over time was performed before phase calculations. CPIs for the group of 40 subjects were calculated according to Equation 3. Random number generator was used to select the subjects from which the time-series were taken. This process was repeated 10000 times and the CPIs were calculated each time. Consequently, 10000 numbers were obtained (for each CP and each scale). These numbers were used to construct the null distributions of each CP and each frequency. The distributions for the different CPs and different frequencies were approximately identical and therefore averaged together. Supplementary Figure 4A shows this average CPI’s null distribution. The distribution implies that CPI of 0.35 corresponded to an uncorrected voxel-level of p = 0.0001. This statistical cutoff was further used in all calculations.

#### PLI null distributions

To define the significance of the pairwise coherences used for comparison with the circular pattern analysis (see “Pairwise coherences of the visual system” section under results), we used the Phase Lag Index (PLI)^22,23^ since it is more similar to the CPI than the phase locking value. Similarly to the CPI, significance was defined from the PLI’s null distributions that were calculated for each scale using permutation tests. The null distributions were calculated by using two uncoupled BOLD signals that were the average BOLD signals from two different visual AAL regions in different subjects (no randomization was used on these regions). Averaging over time of the wavelet coefficients was performed before PLI calculations. PLIs for the group of 40 subjects were calculated for each scale by the following equation:

5 $$PL{I}_{\omega }^{i,j}=\frac{1}{N}|{\sum }_{n=1}^{N}sign({\rm{\Delta }}\phi )|$$

with Δ*φ* the phase-difference between the two BOLD signals of subject *n* at a specific time and frequency window. Random number generator was used to select patients from which time-series were taken. This process was repeated 10000 times and the PLIs were calculated each time. Consequently, 10000 numbers were obtained (for each scale). These numbers were used to construct the null distributions. The distributions for different scales were approximately identical and therefore were averaged together. Supplementary Figure 4B shows this average distribution. The distribution implies that PLI of 0.55 corresponded to an uncorrected voxel-level of p=0.0001. This statistical cutoff was used in the calculations.

#### 3-seed-CPI^k^-SPMs

To obtain the statistical threshold of the *3-seed-CPI*^*k*^*-SPMs* used in the circular-pattern calculations, a CPI uncorrected voxel-level p < 0.0001 and cluster-level threshold of 1000 voxels were used. This cluster size was chosen to ensure a corrected false discovery rate (FDR), given the number of multiple comparisons. Similar to fMRI analysis, the FDR corrected threshold was p < 0.001 which also correct for the multiple *3-seed-CPI*^*k*^*-SPMs* calculations.

#### Seed-PLI-SPMs

To obtain the statistical threshold of the *seed-PLI-SPMs* used in the pairwise coherence calculations, a PLI uncorrected voxel-level p < 0.0001 with a cluster-level threshold of 1000 voxels were used. Together this gave a threshold of p < 0.001, FDR corrected for multiple comparisons.

#### Permutation based non-parametric tests for the number of networks in the dorsal system

To test for differences in the number of networks between the right and left hemispheres, permutation based non-parametric statistics were used. For these comparisons, 50 pairs of pseudo groups each of 40 subjects were constructed. Each pseudo group included *3-seed-CPI*^*k*^*-SPMs* calculated with seeds either in the left or right hemisphere (termed hereinafter “left SPM”, “right SPM”). *3-seed-CPI*^*k*^*-SPMs* were calculated for seed combinations 1, 3 and 4. No tests were performed for seed combination 2 since the difference between left and right dorsal systems for this seed combination was neglectable. The number of left SPMs, within the group of 40 SPMs, was randomly selected with a distribution centered at 20. The number of right SPMs was 40 minus the number of the left SPMs. Pairs of such pseudo groups were constructed such that one group included N left SPMs and M right SPMs, while the other group included 40 minus N left SPMs and 40 minus M right SPMs, with no overlap of subjects between the groups. *3-seed-CPI*^*k*^*-SPMs* of these pseudo groups were calculated using the same calculations and statistical threshold as used in the actual data. The number of networks for each CP and scale was calculated for each pair. These yielded 50 sets of numbers (one set for each pair). The mean and standard deviation (SD) of the differences in the numbers of networks between the pseudo group pairs were calculated. The significant level was defined as mean ± 2*SD estimated at p < 0.05.

As expected, only networks of CP no. 4 (see results) were found significant in these calculations. Significant differences of the number of networks in the dorsal system between the left and right hemispheres were found for seed combinations 3 and 4.

## Electronic supplementary material

Supplementary Figures

## Data Availability

Data is available to anyone upon request.
