# Supplementary material for: Characterizing directed functional pathways in the visual system by multivariate nonlinear coherence of fMRI data
Source: Sci Rep. 2018 Nov 5;8:16362. doi: 10.1038/s41598-018-34672-5 (PMC6218499; doi:10.1038/s41598-018-34672-5)
Supplement: Supplementary file 1 — Supplementary Figures [file 41598_2018_34672_MOESM1_ESM.docx]

**Supplementary information**

Supplement to:

“Characterizing directed functional pathways in the visual system by multivariate nonlinear coherence of fMRI data”

Gadi Goelman, Rotem Dan and Tarek Keadan

**List of contents**

- Supplementary Figure 1: The four seeds used in the *3-seed-CP^k^-SPM* calculations.
- Supplementary Figure 2: *Seed-PLI-SPMs* of the secondary visual cortex area (V2) seeds.
- Supplementary Figure 3: Bivariate and multivariate network’s strength of the ventral and dorsal systems.
- Supplementary Figure 3: Null distributions of the circular pattern index (CPI) and the phase locking index (PLI).


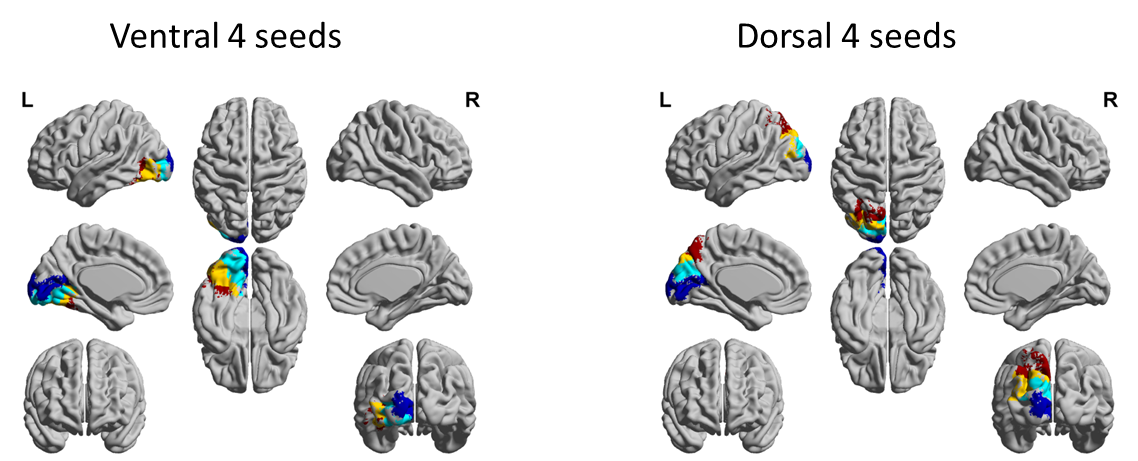


Supplementary Figure 1: The four seeds used in the *3-seed-CP^k^-SPM* calculations.

**V1** - dark blue; **V2** - light blue; **V3** - yellow; **FS** - (ventral) or SP- (dorsal) red.

Supplementary Figure 2: Seed-PLI-SPMs of the secondary visual cortex area (V2) seeds. Statistical parametric maps of pairwise coherences with coherence estimated by the Phase Lag Index (PLI) are presented. V2 seeds of the ventral (upper panel) and dorsal (lower panel) systems and of the left (left side) and right (right side) hemispheres were used. Thresholds of p<0.001 FDR corrected was used.


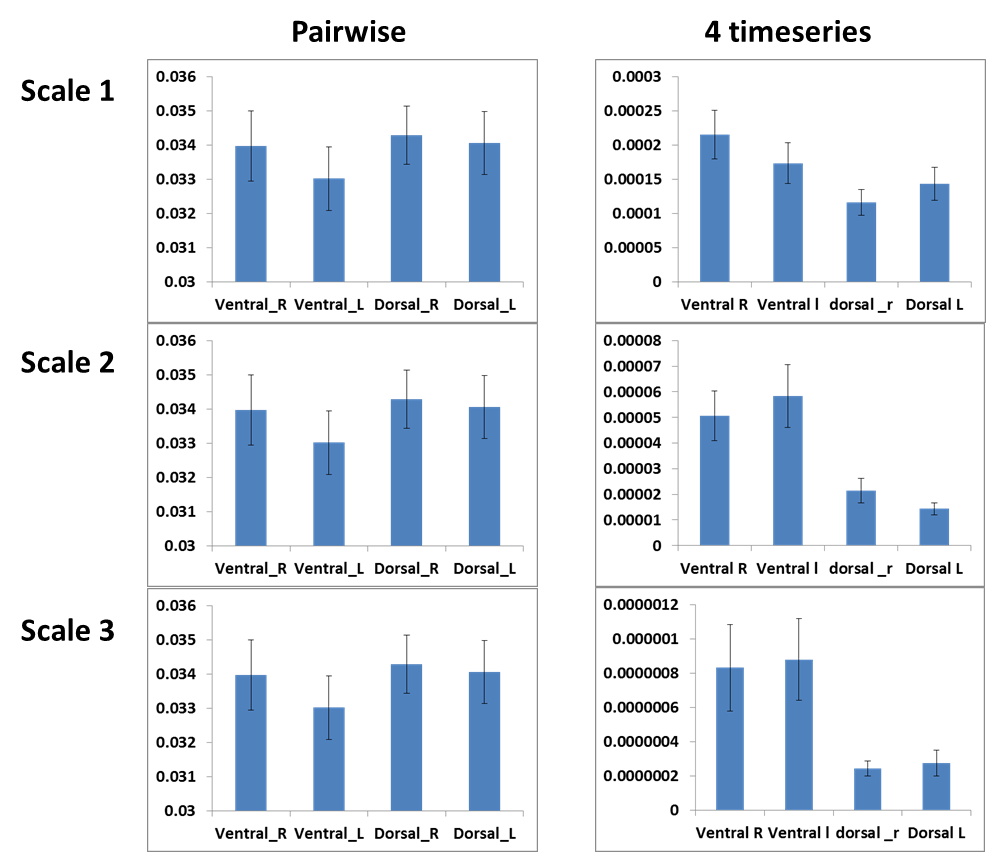


Supplementary Figure 3: Network coherence strength (amplitude) for bivariate pairwise and multivariate 4 timeseries analyses as function of frequency scale. **Left** – The average (±SE) of the pairwise coherence strength over all voxels of the V2_ventral and V2_dorsal seeds of the left and right hemispheres for three frequency scales. **Right** – The average (±SE) of the multivariate 4-timeseries analyses calculated using R1=V2_ventral, R2=V3_ventral, R3=FG for the ventral stream and R1=V2_ventral, R2=V3_dorsal, R3=SP for the dorsal stream of the left and right hemispheres. Note that the amplitude values, for the bivariate and the multivariate analyses, changed approximately as 1/f, as expected.

Supplementary Figure 4: Null distributions of the circular pattern index (CPI) and the phase lag index (PLI). **A**. Null distribution of CPI, averaged over all frequency scales and over all circular patterns. Averaging was done since all distributions were about equal. CPI of 0.35 corresponding to p<0.0001 was used. **B**. Null distribution of PLI averaged over all frequency scales. PLI of 0.55 corresponding to p<0.0001 was used. Distributions were obtained by permutation-based non-parametric tests with uncoupled time-series using 10000 trials.
